# Development of a “Rapid-Crypto Colorimetric LAMP Test” to Detect Cryptosporidiosis in Feces of Newborns Calves

**DOI:** 10.1007/s11686-023-00791-x

**Published:** 2024-02-15

**Authors:** Muhammet Karakavuk, Hüseyin Can, Şengül Can, Tuğba Karakavuk, Mert Döşkaya, Aysu Değirmenci Döşkaya

**Affiliations:** 1 Ege University, Ödemiş Vocational School, Ödemiş, İzmir, Türkiye; 2 Ucyıldız Biotechnology and Veterinary Services, Yunusemre, Manisa, Türkiye; 3 Ege University, Vaccine Development Application and Research Center, Bornova, İzmir, Türkiye; 4 Ege University, Faculty of Science, Department of Biology Molecular Biology Section, Bornova, İzmir, Türkiye; 5 Research Entrepreneurship and Innovation Coordination Center, Manisa Celal Bayar University, Yunusemre, Manisa, Türkiye; 6 Ege University, Faculty of Medicine, Department of Parasitology, Bornova, İzmir, Türkiye

**Keywords:** *Cryptosporidium *spp., Loop-mediated isothermal amplification, LAMP, *18S rRNA*, RT-PCR, Calf

## Abstract

**Background:**

Cryptosporidiosis is a disease that causes major intestinal damage in humans and animals. The causative agents of the disease are *Cryptosporidium* species. In newborn calves, diarrhea can lead to death, resulting in significant economic losses for the farms. Therefore, accurate, rapid, and cost-effective diagnosis of the disease is very important.

**Material and methods:**

In this study, a novel colorimetric loop-mediated isothermal amplification (LAMP) test named “Rapid-Crypto Colorimetric LAMP test” targeting *Cryptosporidium* spp. *18S rRNA* gene was developed to detect cryptosporidiosis in the feces of newborn calves. The analytical sensitivity of the test was determined by plasmid controls. Clinical sensitivity was determined using the feces of 127 calves collected from farms in İzmir and Manisa provinces. All of the samples were also investigated with Real-Time PCR targeting the *Cryptosporidium* spp. *COWP* gene. Cross-reactivity was tested using the DNA of other parasites and bacteria.

**Results:**

According to the results, the analytical sensitivity of the “Rapid-Crypto Colorimetric LAMP test” was found as 1 copy plasmid/reaction. When the results were compared with the Real-Time PCR test, the sensitivity of the “Rapid-Crypto Colorimetric LAMP test” was 100% and the specificity was 97.4%. The test did not cross-react with other parasites and bacteria.

**Conclusion:**

The “Rapid-Crypto Colorimetric LAMP test” developed in this study provides an advantage in the diagnosis of *Cryptosporidium* spp. in calf stool samples since it can be applied in basic laboratories or in the field, does not require experienced personnel, and has high sensitivity. Moreover, diagnosis can be made with the naked eye without using any device.

**Supplementary Information:**

The online version contains supplementary material available at 10.1007/s11686-023-00791-x.

## Introduction

*Cryptosporidium* spp. are zoonotic protozoan parasites that infect humans and animals [1]. Cryptosporidiosis is mainly presented by chronic diarrhea, especially in people with weak immune systems [2]. The disease causes huge economic losses by affecting the intestines of cattle, sheep, and goats. *Cryptosporidium* spp. can also be detected in the conjunctiva, respiratory system, and bursa fabricius of poultry [3].

The most common species in cattle are *Cryptosporidium parvum*,* C. andersoni*,* C. bovis*, and *C. ryanae.* Although cryptosporidiosis is mainly asymptomatic in adult cattle, it can cause death in neonatal calves due to diarrhea. While *C. parvum* is highly pathogenic in neonatal calves,* C. bovis* and *C. ryanae* can also cause clinical diarrhea [4]. Other species rarely produce clinical symptoms in calves [5].

One of the most important causes of newborn calf deaths is diarrhea. The main causative agents responsible for calf diarrhea are *Cryptosporidium* spp., *Escherichia coli*, *Salmonella* spp., rotavirus, and coronavirus [6]. There are studies reporting from Europe that 30% of calf diarrhea is caused by *Cryptosporidium* spp. [7].

Treatment costs increase because of diarrhea caused by cryptosporidiosis in calves. In addition, malabsorption due to intestinal damage leads to significant yield loss. As a result of diarrhea and malabsorption, feed utilization rate decreases in calves and target live weight and milk yield may not be reached in farms [8].

Microscopy, serological, and molecular tests are being used to diagnose the cryptosporidiosis in calves and cattle. Special staining methods, such as Ziehl–Neelsen and Kinyoun acid fast, are used in microscopic diagnosis [8]. Serological tests are based on the detection of antigen or antibody in the stool [9]. In molecular diagnosis, conventional PCR, nested PCR, and Real-Time PCR are used most frequently, and *18S rRNA, gp60, COWP, hsp70* and *β-tubulin* are most frequently targeted genes during diagnosis [10].

Loop-mediated isothermal amplification (LAMP) is a nucleic acid amplification technique with high sensitivity and specificity. It can be applied quickly, easily, and is suitable for use in field conditions since it can operate at a single temperature. In addition, there is no need for advanced laboratory equipment and highly experienced personnel during the application of the LAMP technique [11]. For these reasons, LAMP can be a very useful diagnostic tool to detect *Cryptosporidium* spp. in calves under field conditions.

LAMP tests have been designed to detect *Cryptosporidium* spp. in different samples, such as water and feces samples [1] cattle, sheep and horse feces samples [12], water samples [13], wastewater samples [14], human feces samples [15–17], and cattle and water buffalo feces samples [18].

Hence, in this study, we aimed to develop a novel colorimetric LAMP test named “Rapid-Crypto Colorimetric LAMP test” to detect *Cryptosporidium* spp. *18S rRNA* gene in feces samples of newborn calves, for the first time. Importantly, in this novel test, diagnosis can be achieved with the naked eye without using any device. The analytical performance of this test was evaluated using control plasmids and the clinical sensitivity of the “Rapid-Crypto Colorimetric LAMP test” was determined using feces samples of newborn calves. The results were compared with the Real-Time PCR targeting *Cryptosporidium* spp. *COWP* gene.

## Materials and Methods

### Samples and DNA Isolation

**Fig. 1:**
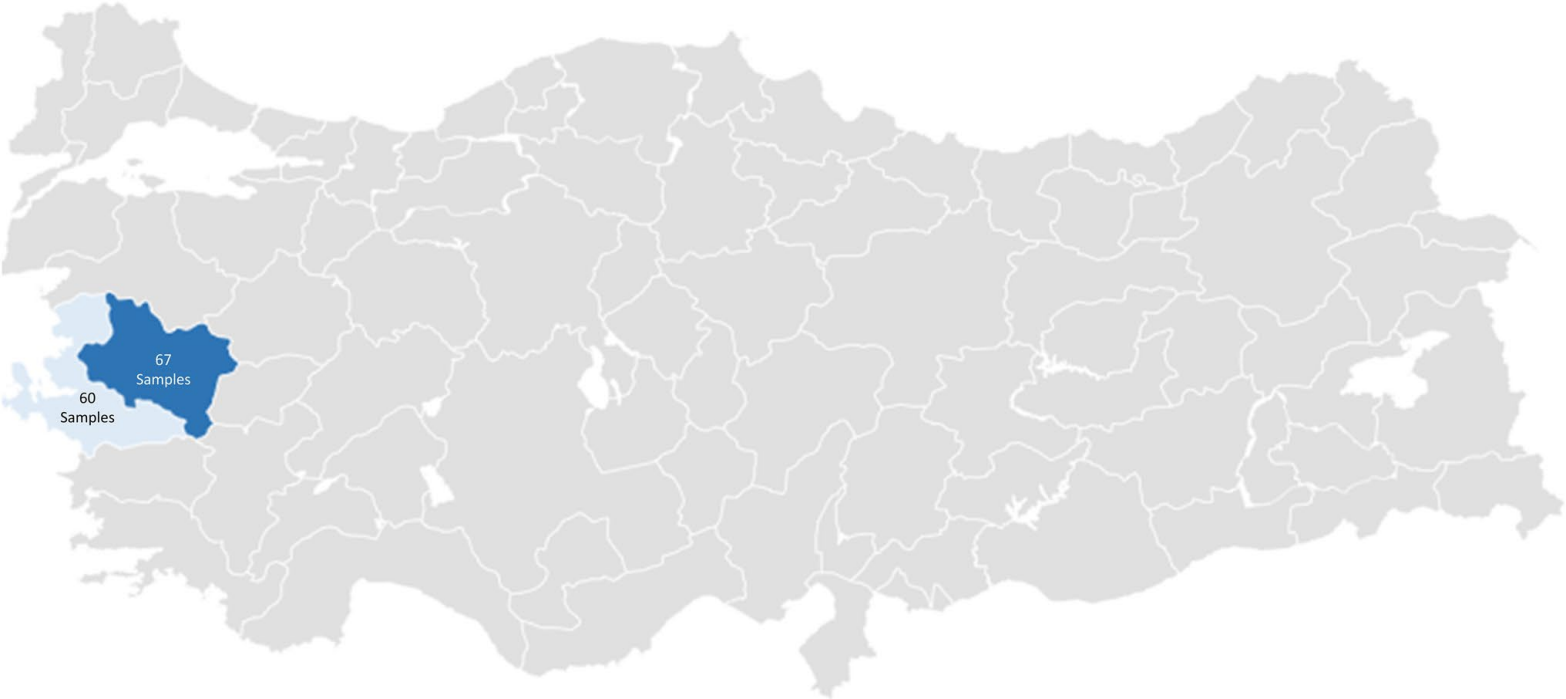
Geographic map of the study area

Feces samples were collected from 127 calves between 2020 and 2021 from farms located in İzmir and Manisa provinces of Türkiye. Feces samples were collected from calves under 1 year of age with diarrhea or the ones who had previously diagnosed with diarrhea. The 67 stool samples were obtained from Manisa province, and the 60 stool samples were obtained from Izmir province (Fig. 1). The RTA DNA Isolation Kit from Stool (İstanbul, Türkiye) was used to extract genomic DNA from feces samples with minor modification [19]. Briefly, 100 mg of feces was added to the purple-capped sample preparation tube containing STL solution (inhibitor breaker) and vortexed at high speed for 30 s. The top cap of the sample preparation tube was opened, and the liquid sample was transferred to a 2 ml centrifuge tube. Samples were incubated at 95 °C for 20 min. After incubation, the samples were centrifuged at 18,000×*g* for 3 min. The supernatant was transferred to a new 1.5 ml centrifuge tube and centrifuged again. The supernatant was transferred to a new 1.5 ml centrifuge tube, 25 µl proteinase K was added, and vortex and spin-down was performed. Then, 500 µl of solution B was added, vortexed for 5 s, and incubated at 70 °C for 10 min. The 650 µl absolute ethanol was added to the samples and vortex spin-down was performed. The samples were added to the filter tubes of the kit and centrifuged at 18,000×*g* for 30 s. Then, the filter tubes were washed with 700 µl of W1 and W2 solutions, respectively, by centrifuging for 30 s at 18,000×*g*. After the filter tubes were opened and kept at 70 °C for 5 min, the empty filter tube was centrifuged at 18,000×*g* for 1 min. The filter tubes were placed on new 1.5 ml centrifuge tubes, 100 µl of solution E was added and waited at room temperature for 3 min. Finally, the tube was centrifuged at 18,000×*g* for 1 min and 30 s, respectively. The obtained genomic DNA was stored at -20 °C until Real-Time PCR was performed.

### Design of The LAMP Primer Set Targeting *Cryptosporidium* spp. *18S rRNA* Gene

Specific primer set targeting the *Cryptosporidium* spp. *18S rRNA* gene (GenBank No: AF513227.2) was designed using PrimerExplorer V5 software (http://primerexplorer.jp/lampv5e/index.html) (Table 1). The designed primers were confirmed to be gene-specific by the National Center for Biotechnology Information (NCBI) BLAST program (https://blast.ncbi.nlm.nih.gov/Blast.cgi).

**Table 1:** Primer sets of “Rapid-Crypto Colorimetric LAMP test”

Lamp test	Primer type	Primer length (nt)	Sequence (5′– 3′)	Amplicon size	Target
“Rapid-Crypto Colorimetric LAMP test”	F3	20	CCACTAGAGGAGCTACTGTA	207 bp	*Cryptosporidium* spp. *18S rRNA*
	B3	18	TGACAAGCTACAACACGT		
	FIP	48	AGGTGAGGGTTTTCTACATCACTAT-ATTGGAACAAGCAAATTCTATGG		
	BIP	43	ATGGGTTGGGATTATCCTAAATGTG-TGCGAGCAAGAACAAGTG		
	LF	25	CAGTTTTTAACATGTTGTGCCAACC		
	LB	21	TAGAGCCATGCCTAACATGCT		

### Construction of Positive Control Plasmid

Initially, the targeted *18S rRNA* gene region was isolated from the DNA sample of a *Cryptosporidium*-positive feces which was previously confirmed by PCR using external primers F3 and B3 (Table 1) and previously described protocol [20]. The feces sample was obtained from a < 2-month-old calf presenting with diarrhea and *Cryptosporidium* oocyst were detected in the calf’s stool by microscopy, which was further confirmed by Real-Time PCR and nested PCR. Next, the PCR-positive strain was identified as *C. parvum* by sequencing [20]. During PCR, the 25 ml amplification reaction included 5 ng template DNA, the F3 and B3 primers (0.4 mM each), 1.25U Taq DNA Polymerase (ThermoScientific), 200 mM dNTPs, 1 mM MgCl_2_, and 1 × Taq reaction buffer. The PCR amplification reaction was performed using the following calculated protocol: 10 min initial denaturation step at 95 °C, followed by 35 cycles of 30 s at 95 °C, 30 s at 53 °C, and 30 s at 72 °C, and a final extension of 10 min at 72 °C. The PCR product with a final size of 207 bp was visualized by 1% agarose gel electrophoresis.

Later, the PCR product was purified with the PCR purification kit (QIAquick^®^ PCR Purification Kit, Qiagen, Germany) and cloned into the pCR 2.1-TOPO vector (Thermoscientific, USA) according to the manufacturers’ protocol. Briefly, 1 μl of purified PCR product, 1 μl of vector, 1 μl of salt solution, and 3 μl of molecular distilled water were mixed and incubated at room temperature for 20 min. Then, 50 μl of TOP10 *E. coli* competent cells were added to the mixture and incubated on ice for 20 min. After incubation, the mixture was heat-shocked at 42 °C for 30 s and 250 µl of S.O.C medium was added quickly and incubated at 225 rpm at 37 °C for 1 h. Cells were then seeded on LB-Agar plates containing 50 µg/ml kanamycin and incubated overnight at 37 °C. Next day, a single colony was selected and inoculated into 3 ml of LB medium containing 50 µg/ml kanamycin and incubated overnight at 37 °C at 225 rpm. Plasmid purification from the obtained colonies was carried out in accordance with the kit protocol (QIAprep Spin Miniprep Kit, Qiagen, Germany). The presence of *18S rRNA* gene in pCR 2.1.-TOPO vector (named pCR 2.1-Crypto18SrRNA) was confirmed by 1% agarose gel electrophoresis and sequencing [20]. Thereafter, the concentration of pCR 2.1-Crypto18SrRNA in purified sample was determined by Nanodrop spectrophotometer (Thermoscientific, USA) and the amount of copy plasmid in purified sample was calculated using the following formula “Number of copies = (DNA amount in ng × [6.02 × 10^23^]) / (pCR 2.1-Crypto18SrRNA plasmid length in bp) × [1 × 10^9^] × 650) [21].

### Development of LAMP Test

Development of “Rapid-Crypto Colorimetric LAMP test” was carried out with WarmStart^®^ Colorimetric LAMP 2 × Master Mix kit (NEB, USA). A 15 μl reaction consisted of 7.5 μl of Master Mix, 1 μl of control plasmid (10^3^ copy plasmid/reaction), and 1.75 μl primer mix [containing inner primers outer primers F3 and B3 (0.1 μM each), loop primers LF and LB (0.2 μM each), FIP and BIP (0.8 μM each)]. “Rapid-Crypto Colorimetric LAMP test” was incubated at 58 °C for 60 min in a Dry Bath Incubator (Major Science, USA). In order to determine the optimal incubation time, LAMP tubes were incubated at 60 °C for 5 to 70 min [22]. The optimum reaction temperature for the LAMP test was determined as 58 °C in our previous study [23].

To assess of analytical sensitivity of “Rapid-Crypto Colorimetric LAMP test”, pCR 2.1-Crypto18SrRNA plasmid with an 8.39 × 10^13^/μl concentration was initially diluted to 1 × 10^13^/μl and then diluted with distilled water to make positive controls containing 10^6^–10^5^–10^4^–10^3^-10^2^–10–1–10^−1^ plasmid copies/reaction. Sterile DNase- and RNase-free distilled water was used as negative control.

### Cross-Reactivity of “Rapid-Crypto Colorimetric LAMP Test”

The cross-reactivity of the “Rapid-Crypto Colorimetric LAMP test” was determined using DNA samples belonging to *Toxoplasma gondii* Ankara strain [24]. *Leishmania infantum* (MHOM/TN/80/IPT1 strain), *Plasmodium vivax* (ATCC MR4-ATCC)*, Entamoeba histolytica* (ATCC 30459D)*, Acanthamoeba* spp. (*Acanthamoeba* sp. strain DP/5A with T4 genotype), *Giardia intestinalis* (microscopy confirmed positive sample), *Escherichia coli* (BL21 strain)*, P. jirovecii* (PCR confirmed positive control sample) [25], and *Trichomonas vaginalis* (microscopy confirmed positive control sample) [26]. These DNA samples were investigated by “Rapid-Crypto Colorimetric LAMP test” as described in Sect. "Development of LAMP Test".

### Real-Time PCR

The 151 bp region of *Cryptosporidium* spp. *COWP* gene (GenBank accession number: AF248743.1) was targeted by Real-Time PCR. The hydrolysis probe was 5’- TGCCATACATTGTTGTCCTGACAAATTGAAT -3’-BHQ (31nt, cowp-P702, labeled at the 3’ end with FAM), and the primers were 5’-CAAATTGATACCGTTTGTCCTTCTG-3’ (25nt, cowp-P702 forward primer) and 5’-GGCATGTCGATTCTAATTCAGCT-3’ (23nt, cowp-P702 reverse primer). PCR reaction with a 20 µl final volume consisted of 1 × LightCycler Taqman Master mix, 5 µl DNA template or controls, 0.5 µM from each primer, and 0.1 µM probe. The amplification reaction was performed as follows: 10 min initial denaturation step at 95 °C, followed by 45 cycles of 10 s at 95 °C, 15 s at 55 °C, and 15 s at 72 °C [19, 27].

### Clinical Sensitivity of “Rapid-Crypto Colorimetric LAMP Test”

The clinical sensitivity of the developed “Rapid-Crypto Colorimetric LAMP test” was determined by comparing the LAMP test results of the collected stool samples with the Real-Time PCR test targeting *COWP* gene [19].

## Results

### Primer Blast Results

The gene fragments targeted by LAMP primers in *18S rRNA* gene were screened with the NCBI BLAST program and it was found that each gene fragment was 100% similar to *Cryptosporidium* species. Any similarity was not detected with other pathogens (Supplementary material).

### Development of “Rapid-Crypto Colorimetric LAMP Test” Using Control Plasmids

**Fig. 2:**
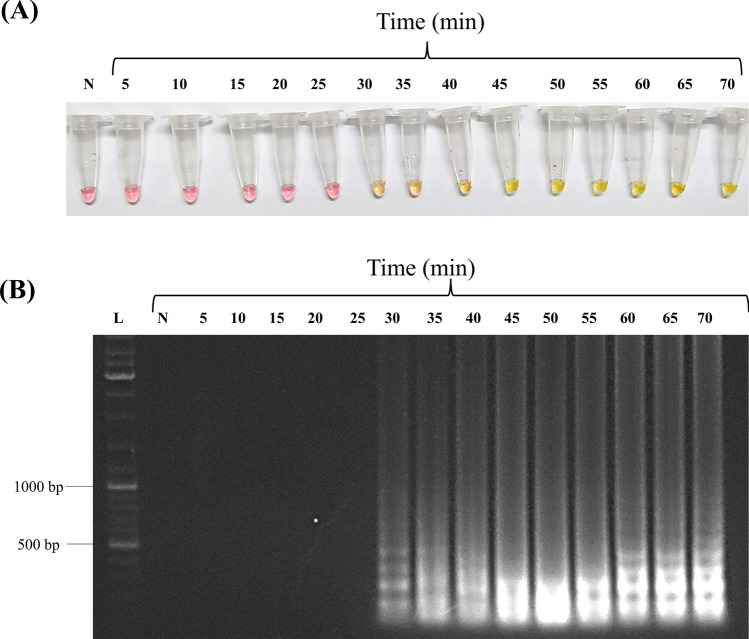
Optimization of incubation time for the *“*Rapid-Crypto Colorimetric LAMP test*”*. Each tube contained 10^3^ copy plasmid/reaction. The sample was incubated at 60 °C for 5 to 70 min at 5 min intervals to determine the optimum incubation time. **A** Colorimetric LAMP results indicated by dye and visualized by eye **B** Amplified DNA products were analyzed by 1% agarose gel electrophoresis. *L* DNA ladder, *N* Negative

As shown in Fig. 2, the “Rapid-Crypto Colorimetric LAMP test” product can detect *18S rRNA* gene region starting at 30 min both by analyses of test tubes by naked eye (Fig. 2A) which was confirmed by agarose gel electrophoresis results (Fig. 2B). The most significant color change (i.e., LAMP product) was detected in tube incubated for 50 min (Fig. 2).

### Analytical Sensitivity of “Rapid-Crypto Colorimetric LAMP Test”

**Fig. 3:**
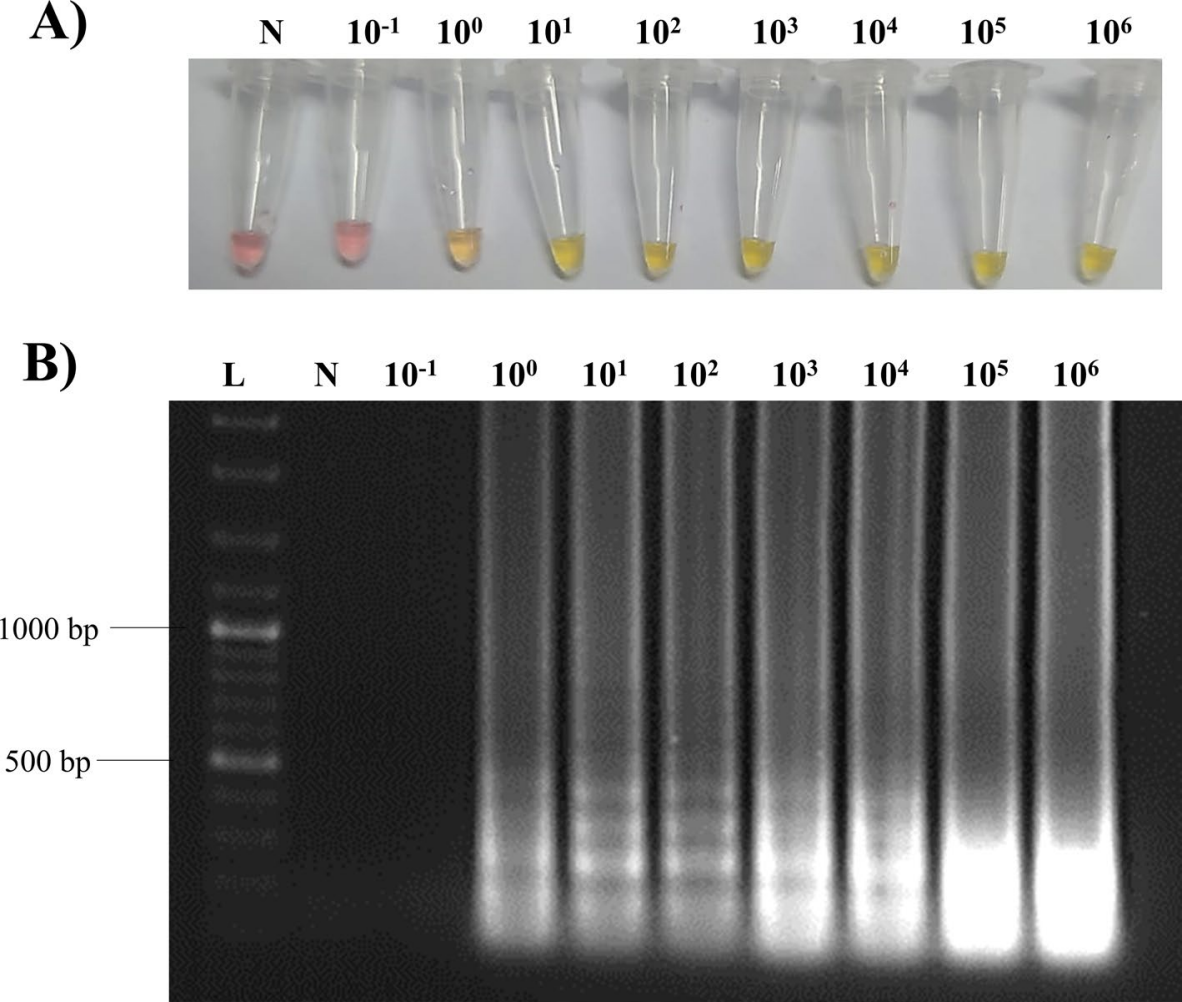
Analytical sensitivity of the “Rapid-Crypto Colorimetric LAMP test”. **A** Results of the “Rapid-Crypto Colorimetric LAMP test” as detected by naked eye **B** Amplified DNA products of LAMP RE assay were analyzed by 1% agarose gel electrophoresis

Analytical sensitivity of “Rapid-Crypto Colorimetric LAMP test” was determined using serially diluted pCR 2.1-Crypto18SrRNA plasmids. According to the results, the analytical sensitivity of “Rapid-Crypto Colorimetric LAMP test” was 1 copy plasmid/reaction (Fig. 3).

### Clinical Sensitivity of “Rapid-Crypto Colorimetric LAMP Test”

As a result of the Real-Time PCR test targeting, the *COWP* gene of 127 calf feces samples, 49 positive (38.58%) were positive, and 78 samples were negative. “Rapid-Crypto Colorimetric LAMP test” detected 51 positive samples among 127 calf feces samples (40.15%). If we use Real-Time PCR targeting *COWP* gene as a reference method, the clinical sensitivity of “Rapid-Crypto Colorimetric LAMP test” was 100%, whereas specificity was 97.4% (Table 2). The two samples detected positive by LAMP and negative by Real-Time PCR were collected from calves with diarrhea.

**Table 2:** Sensitivity, Specificity, PPV (Positive Predictive Value), NPV (Negative Predictive Value), and accuracy values of LAMP assay using Real-Time PCR targeting *COWP* gene of *Cryptosporidium* as reference test

Methods	Sensitivity (%)	Specificity (%)	PPV (%)	NPV (%)	Accuracy
Real-time PCR	100	100	100	100	100
LAMP	100	97.4	96	97.5	96.9

### Cross-Reactivity of “Rapid-Crypto Colorimetric LAMP Test”

**Fig. 4:**
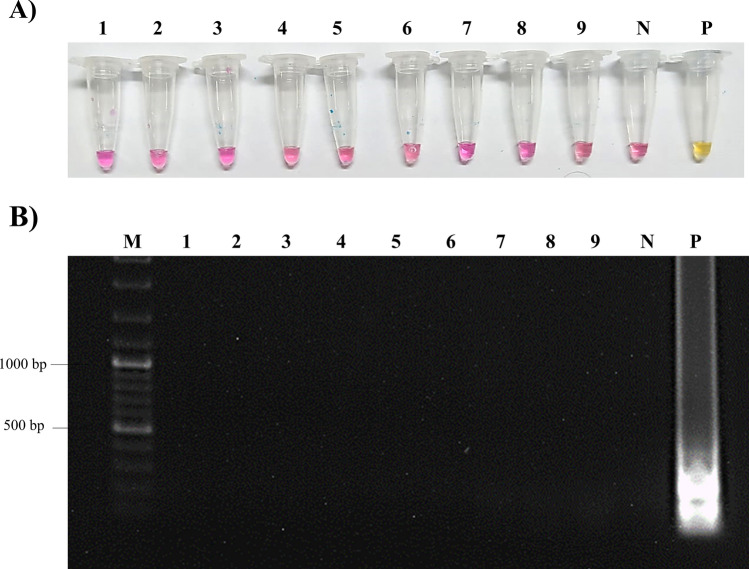
Cross-reactivity of the “Rapid-Crypto Colorimetric LAMP test”. **A** The “Rapid-Crypto Colorimetric LAMP test” as detected by naked eye **B** Amplified DNA products of “Rapid-Crypto Colorimetric LAMP test” were analyzed by 1% agarose gel electrophoresis. 1: *T. gondii*, 2: *P. vivax*, 3: *E.* *histolytica*, 4: *L. infantum*., 5: *Acanthamoeba* spp., 6: *G. intestinalis*, 7: *E. coli*, 8: *P. jirovecii* 9: *T. vaginalis*. *L* DNA ladder, *N* Negative, *P* Positive

Cross-reactivity of “Rapid-Crypto Colorimetric LAMP test” was determined using DNA samples belonging to *Toxoplasma gondii, Plasmodium vivax, Entamoeba* spp.*, Leishmania infantum, Acanthamoeba* spp*., Giardia intestinalis, Escherichia coli, Pneumocystis jirovecii, *and* Trichomonas vaginalis.* According to the results, any positive LAMP reaction was not observed in any of the parasites and bacteria analyzed (Fig. 4).

## Discussion

Calf health is vital for sustainable herd health all over the world. Accordingly, a calf birth from each cow is aimed each year for an economical farm management [28]. Diarrhea caused by both infectious agents and other factors  in newborn calves’ results with great economic losses worldwide. *Escherichia coli*, *Cryptosporidium* spp., coronavirus, and rotavirus are the most common neonatal diarrhea causing pathogenic agents in calves [29–31]. In many studies, it has been stated that frequency of diarrhea in calves is associated with the frequency of *Cryptosporidium* spp. [32].

The prevalence of cryptosporidiosis in cattle is around 30% worldwide and because of this high prevalence rapid, accurate, and economical diagnosis of *Cryptosporidium* spp. is very important in maintaining and managing herd health [33]. The diagnosis of *Cryptosporidium* spp. in calves is achieved by microscopy, serological, or molecular tests. Microscopy has disadvantages such as the need for experienced personnel and it has low sensitivity [19]. In serological diagnosis, the sensitivity is also low [34]. During molecular diagnosis using methods, such as PCR, nested PCR, or Real-Time PCR targeting *Cryptosporidium* spp., experienced personnel is required and high-cost laboratory setup is an important disadvantage, and for these reasons, their application under field conditions is very limited.

In this study, a “Rapid-Crypto Colorimetric LAMP test” was developed which can achieve rapid, accurate, and economical diagnosis of *Cryptosporidium* spp. in calves. The test does not require advanced laboratory equipment and has a sensitivity of 1 copy of plasmid/reaction (Fig. 3). In addition, it did not cross-react with other bacteria, parasites, or viruses using bioinformatic analyzes (Fig. 4 and Supplementary material). We used Real-Time PCR targeting *COWP* gene as a reference method in this study because the *COWP* gene has high sensitivity and specificity in the diagnosis of *Cryptosporidium* spp. [35]. Accordingly, the clinical sensitivity and the specificity of “Rapid-Crypto Colorimetric LAMP test” were 100% and 97.4%, respectively (Table 2). The remaining two samples detected positive by LAMP and negative by Real-Time PCR were collected from claves with diarrhea.

There are a limited number of *Cryptosporidium* LAMP studies. In a study, the *Cryptosporidium parvum gp60* gene-specific LAMP test was developed and its sensitivity was determined as 1 oocyst/reaction. The developed test was found to be more sensitive than PCR. In addition, the developed test was studied with environmental and feces samples that were categorized by the fluorescent antibody test and was found to be compatible with the fluorescent antibody test [1] Among the 270 stool samples that were negative with nested PCR, 79 samples were found positive (29.95%) with LAMP test targeting the *SAM-1* gene region [12]. In a previously developed LAMP test targeting other segments of *18S rRNA* gene, the detection limit was determined as 6 × 10^–3^ oocysts, and it was found 3.5 times more sensitive than microscopy in environmental samples [13]. The analytical sensitivity of an LFD-LAMP test targeting the *SAM-1* gene was 10 oocysts/ml and it was shown that the test is 10 times more sensitive than the nested PCR test [17]. The sensitivity of the *SAM-1* LAMP test modified by adding uracil DNA glycosylase (UDG) was found to be 10 oocysts/reaction [16]. LAMP test targeting *Cryptosporidium parvum* *gp60* gene was performed with cattle and buffalo feces samples that were *Cryptosporidium*-positive with Kinyoun acid fast. However, the sensitivity of this test has not been showed [18].

LAMP tests developed in previous studies work with SYBR green, turbidimeter, or gel electroporation methods, and moreover additional devices, such as electrophoresis device, turbidimeter, or UV lamp, are required to interpret the results. The “Rapid-Crypto Colorimetric LAMP test” developed in this study has an important advantage over these approaches by being able to visually diagnose *Cryptosporidium* spp. with naked eye.

In our previous publication [19], there were 6 positive and 25 negative *Cryptosporidium* spp. water samples collected from water resources as detected by Real-Time PCR targeting *COWP*. The “Rapid-Crypto Colorimetric LAMP test” developed in this study was applied to these samples, and it showed 100% agreement (Data not shown). For this reason, it was thought that the “Rapid-Crypto Colorimetric LAMP test” developed for *Cryptosporidium* spp. in this study can also work appropriately with water samples, which are important sources of cryptosporidiosis.

## Conclusion

As a result, rapid, accurate, and economical diagnosis of *Cryptosporidium* spp. in feces samples of calves have utmost importance for herd health. “Rapid-Crypto Colorimetric LAMP test” is a diagnostic method that can be applied both by clinician veterinarians and by personnel with basic laboratory knowledge (such as veterinary technicians and biologists) working in basic laboratories established in farms or in the field. With the definitive and rapid diagnosis of the cryptosporidiosis, treatment costs and loss of yield will be reduced, and it will make a positive contribution to both the farm and the country’s economy. 

### Supplementary Information

Below is the link to the electronic supplementary material.

Supplementary material PDF (444 KB)

## Data Availability

All data supporting the findings of this study are available within the paper and its Supplementary Information.

## References

[1] Karanis P, Thekisoe O, Kiouptsi K, Ongerth J, Igarashi I, Inoue N (2007). Development and preliminary evaluation of a loop-mediated isothermal amplification procedure for sensitive detection of *Cryptosporidium oocysts* in fecal and water samples. Appl Environ Microbiol.

[2] Laurent F, Lacroix-Lamandé S (2017). Innate immune responses play a key role in controlling infection of the intestinal epithelium by *Cryptosporidium*. Int J Parasitol.

[3] Xiao L, Feng Y (2008). Zoonotic cryptosporidiosis. FEMS Immunol Med Microbiol.

[4] Lichtmannsperger K, Harl J, Freudenthaler K, Hinney B, Wittek T, Joachim A (2020). *Cryptosporidium *
*parvum*. Cryptosporidium ryanae, and Cryptosporidium bovis in samples from calves in Austria.

[5] Santín M, Trout JM, Xiao L, Zhou K, Greiner E, Fayer R (2004). Prevalence and age-related variation of *Cryptosporidium* species and genotypes in dairy calves. Vet Parasitol.

[6] Thompson HP, Dooley JSG, Kenny J, McCoy M, Colm LJ, John EM (2007). Genotypes and subtypes of *Cryptosporidium* spp. in neonatal calves in Northern Ireland. Parasitol Res.

[7] Şahal M, Terzi OS, Ceylan E, Kara E (2018). Buzağı İshalleri ve Korunma Yöntemleri.

[8] (2019) Büyükbaş Hayvancılık (Sığırcılık), Tarım Orman Bakanlığı. https://www.tarimorman.gov.tr/HAYGEM/Belgeler/Hayvancılık/BüyükbaşHayvancılık/2019.Yılı/Buyukbas_Hayvan_Yetistiriciligi.pdf

[9] Weber SE, Lippuner C, Corti S, Deplazes P, Hassig M (2016). Clinical epidemiology of cryptosporidiosis in calves. Schweiz Arch Tierheilkd.

[10] Cacciò SM, Thompson RCA, McLauchlin J, Smith HV (2005). Unravelling *Cryptosporidium* and giardia epidemiology. Trends Parasitol.

[11] Nagamine K, Hase T, Notomi T (2002). Accelerated reaction by loop-mediated isothermal amplification using loop primers. Mol Cell Probes.

[12] Bakheit MA, Torra D, Palomino LA, Thekisoe OMM, Mbati PA, Ongerth J (2008). Sensitive and specific detection of *Cryptosporidium* species in PCR-negative samples by loop-mediated isothermal DNA amplification and confirmation of generated LAMP products by sequencing. Vet Parasitol.

[13] Inomata A, Kishida N, Momoda T, Akiba M, Izumiyama S, Yagita K (2009). Development and evaluation of a reverse transcription-loop-mediated isothermal amplification assay for rapid and high-sensitive detection of *Cryptosporidium* in water samples. Water Sci Technol.

[14] Gallas-Lindemann C, Sotiriadou I, Plutzer J, Noack MJ, Mahmoudi MR, Karanis P (2016). Giardia and *Cryptosporidium* spp. dissemination during wastewater treatment and comparative detection via immunofluorescence assay (IFA), nested polymerase chain reaction (nested PCR) and loop mediated isothermal amplification (LAMP). Acta Trop.

[15] Nago TT, Tokashiki YT, Kisanuki K, Nakasone I, Yamane N (2010). Laboratory-based evaluation of loop-mediated isothermal amplification (LAMP) to detect *Cryptosporidium oocyst* and *Giardia **lamblia* cyst in stool specimens. The Japanese J of Clinical Pathology.

[16] Fallahi S, Moosavi SF, Karimi A, Chegeni AS, Saki M, Namdari P (2018). An advanced uracil DNA glycosylase-supplemented loop-mediated isothermal amplification (UDG-LAMP) technique used in the sensitive and specific detection of *Cryptosporidium **parvum*, *Cryptosporidium **hominis*, and *Cryptosporidium **meleagridis* in AIDS patients. Diagn Microbiol Infect Dis.

[17] Mamba TS, Mbae CK, Kinyua J, Mulinge E, Mburugu GN, Nijiru Z (2018). Lateral Flow Loop-Mediated Isothermal Amplification Test with Stem Primers: Detection of *Cryptosporidium* Species in Kenyan Children Presenting with Diarrhea. J Trop Med.

[18] Domingo CYJ, Pascual HG, Mingala CN (2018). Detection of *Cryptosporidium parvum* DNA in fecal samples of infected cattle (Bos indicus) and water buffaloes (bubalus bubalis) in the PHİLİPPİNES using loop mediated isothermal amplification method. Ann Parasitol.

[19] Karakavuk M, Can H, Döşkaya M, Karakavuk T, ErkuntAlak S, Köseoğlu AE (2021). Cryptosporidiosis outbreak on a dairy farm: detection of *Cryptosporidium parvum* as a causative agent in the water source. Pol J Vet Sci.

[20] Karakavuk M, Can H, Karakavuk T, Gül C, Erkunt-Alak S, Gül A (2021). Toxoplasma gondii 529 baz çifti büyüklüğünde tekrar bölgesine (RE) özgü hızlı LAMP testinin geliştirilmesi ve analitik hassasiyetinin belirlenmesi. Ege Tıp Derg.

[21] Morozov VA, Morozov AV, Denner J (2016). New PCR diagnostic systems for the detection and quantification of porcine cytomegalovirus (PCMV). Arch Virol.

[22] Tran DH, Tran HT, Le UP, Vu XD, Trinh TBN, Do HDK (2020). Direct colorimetric LAMP assay for rapid detection of African swine fever virus: A validation study during an outbreak in Vietnam. Transbound Emerg Dis.

[23] Karakavuk M (2021) Cryptosporidium’un LAMP ile Tanısında Floresan ve Kolorimetrik LAMP Kitlerinin Karşılaştırılması. Uluslararasi 22. Parazitoloji Kongresi. pp 199–199. (http://www.hmr.com.tr/Kongre/tr-TR/Home/Page/0?s=KongreProgrami&kongre=parazitoloji-2021)

[24] Deĝirmenci A, Döşkaya M, Caner A, Çiçek C, Korkmaz M, Gürüz Y (2011). Toxoplasma gondii RH Ankara: Production of evolving tachyzoites using a novel cell culture method. Exp Parasitol.

[25] Sürgeç E, Can H, Döşkaya M, Karakavuk M, AtalayŞahar E, DeğirmenciDöşkaya A (2020). Genotyping of Pneumocystis jirovecii isolates obtained from clinical samples by multilocus sequencing: a molecular epidemiology study conducted in Turkey. Arch Microbiol.

[26] Ertabaklar H, Caner A, Döşkaya M, Döşkaya M, Demiştaş LO, Töz S (2011). Comparison of polymerase chain reaction with wet mount and culture methods for the diagnosis of trichomoniasis. Türkiye parazitolojii derg / Türkiye Parazitoloji Derǧ = Acta parasitologica Turc / Turkish Soc for Parasitology.

[27] Taniuchi M, Verweij JJ, Noor Z, Sobuz SU, Van Lieshout L (2011). High throughput multiplex PCR and probe-based detection with luminex beads for seven intestinal parasites. Am J Trop Med Hyg.

[28] Erdem H, Atasever S, Kul E (2007). Milk yield and fertility traits of holstein cows raised at gokhoyuk state farm 1. milk yield traits. Anadolu J of Agricultural Sci.

[29] Lorenz I, Fagan J, More SJ (2011). Calf health from birth to weaning II Management of diarrhoea in pre-weaned calves. Ir Vet J.

[30] Meganck V, Hoflack G, Piepers S, Opsomer G (2015). Evaluation of a protocol to reduce the incidence of neonatal calf diarrhoea on dairy herds. Prev Vet Med.

[31] Ok M, Güler L, Turgut K, Ok Ü, Şen I, Gündüz IK (2009). The studies on the aetiology of diarrhoea in neonatal calves and determination of virulence gene markers of *escherichia** coli* strains by multiplex PCR. Zoonoses Public Health.

[32] Torsein M, Lindberg A, Sandgren CH, Walker KP, Törnquist M, Svensson C (2011). Risk factors for calf mortality in large Swedish dairy herds. Prev Vet Med.

[33] Hatam-Nahavandi K, Ahmadpour E, Carmena D, Spotin A, Bangoura B, Xiao L (2019). *Cryptosporidium* infections in terrestrial ungulates with focus on livestock: A systematic review and meta-analysis. Parasit Vectors.

[34] Danišová O, Halánová M, Valenčáková A, Luptáková L (2018). Sensitivity, specificity and comparison of three commercially available immunological tests in the diagnosis of *Cryptosporidium* species in animals. Braz J Microbiol.

[35] Weinreich F, Hahn A, Eberhardt KA, Feldt T, Sarfo FS, Di Cristanziano V (2022). Comparison of Three Real-Time PCR Assays for the detection of Cyclospora cayetanensis in Stool samples targeting the 18S rRNA gene and the hsp70 gene. Pathogens.

