# Supplementary material for: Development of a “Rapid-Crypto Colorimetric LAMP Test” to Detect Cryptosporidiosis in Feces of Newborns Calves
Source: Acta Parasitol. 2024 Feb 15;69(1):691–9. doi: 10.1007/s11686-023-00791-x (PMC11001726; doi:10.1007/s11686-023-00791-x)
Supplement: Supplementary file 1 — Supplementary material PDF (444 KB) [file 11686_2023_791_MOESM1_ESM.pdf]

An official website of the United States government

Here's how you know

The .gov means it’s official.
Federal government websites often end in .gov or .mil. Before sharing sensitive information, make sure you’re on a federal government site.

The site is secure.
The **https://** ensures that you are connecting to the official website and that any information you provide is encrypted and transmitted securely.

**Job Title**	Nucleotide Sequence ...
**RID**	ANN89W9P016 Search expires on 06-17 15:00 pm
**Program**	BLASTN
**Database**	nt
**Query ID**	lcl|Query_24575
**Description**	None ...
**Molecule type**	dna
**Query Length**	207

Descriptions

Description	Scientific Name	Max Score	Total Score	Query Cover	E value	Per. Ident	Acc. Len	Accession
Cryptosporidium parvum strain KSU-1 small subunit ribosomal RNA genes, partial sequence; internal transcribed spacer 1, putative 5.8S ribosomal RNA gene, and internal transcribed spacer 2, complete sequence; and large subunit ribosomal RNA gene, partial sequence	Cryptosporidium parvum	383	383	100%	5e-102	100.00%	3581	AH006572.2
Cryptosporidium parvum strain HLA 18S ribosomal RNA gene, partial sequence	Cryptosporidium parvum	383	383	100%	5e-102	100.00%	1735	AH008195.2
Cryptosporidium parvum isolate IQ.Cp-15 18S ribosomal RNA gene, partial sequence	Cryptosporidium parvum	383	383	100%	5e-102	100.00%	1319	KT151554.1
Cryptosporidium parvum isolate IQ.Cp-14 18S ribosomal RNA gene, partial sequence	Cryptosporidium parvum	383	383	100%	5e-102	100.00%	1214	KT151552.1
Cryptosporidium meleagridis isolate IQ.Cm-4 18S ribosomal RNA gene, partial sequence	Cryptosporidium meleagridis	383	383	100%	5e-102	100.00%	1487	KT151551.1
Cryptosporidium meleagridis isolate IQ.Cm-3 18S ribosomal RNA gene, partial sequence	Cryptosporidium meleagridis	383	383	100%	5e-102	100.00%	1317	KT151549.1
Cryptosporidium parvum isolate IQ.Cp-13 18S ribosomal RNA gene, partial sequence	Cryptosporidium parvum	383	383	100%	5e-102	100.00%	1251	KT151548.1
Cryptosporidium parvum isolate IQ.Cp-12 18S ribosomal RNA gene, partial sequence	Cryptosporidium parvum	383	383	100%	5e-102	100.00%	1319	KT151547.1
Cryptosporidium parvum isolate IQ.Cp-10 18S ribosomal RNA gene, partial sequence	Cryptosporidium parvum	383	383	100%	5e-102	100.00%	1261	KT151541.1
Cryptosporidium parvum isolate IQ.Cp-9 18S ribosomal RNA gene, partial sequence	Cryptosporidium parvum	383	383	100%	5e-102	100.00%	1261	KT151540.1
Cryptosporidium meleagridis isolate IQ.Cm-2 18S ribosomal RNA gene, partial sequence	Cryptosporidium meleagridis	383	383	100%	5e-102	100.00%	1259	KT151539.1

Description	Scientific Name	Max Score	Total Score	Query Cover	E value	Per. Ident	Acc. Len	Accession
Cryptosporidium meleagridis isolate IQ.Cm-1 18S ribosomal RNA gene, partial sequence	Cryptosporidium meleagridis	383	383	100%	5e-102	100.00%	1317	KT151537.1
Cryptosporidium parvum isolate IQ.Cp-8 18S ribosomal RNA gene, partial sequence	Cryptosporidium parvum	383	383	100%	5e-102	100.00%	1319	KT151536.1
Cryptosporidium parvum isolate IQ.Cp-7 18S ribosomal RNA gene, partial sequence	Cryptosporidium parvum	383	383	100%	5e-102	100.00%	1319	KT151535.1
Cryptosporidium parvum isolate IQ.Cp-6 18S ribosomal RNA gene, partial sequence	Cryptosporidium parvum	383	383	100%	5e-102	100.00%	1203	KT151533.1
Cryptosporidium parvum isolate IQ.Cp-5 18S ribosomal RNA gene, partial sequence	Cryptosporidium parvum	383	383	100%	5e-102	100.00%	1363	KT151531.1
Cryptosporidium parvum isolate IQ.Cp-4 18S ribosomal RNA gene, partial sequence	Cryptosporidium parvum	383	383	100%	5e-102	100.00%	1261	KT151530.1
Cryptosporidium parvum isolate IQ.Cp-3 18S ribosomal RNA gene, partial sequence	Cryptosporidium parvum	383	383	100%	5e-102	100.00%	1213	KT151529.1
Cryptosporidium parvum isolate IQ.Cp-2 18S ribosomal RNA gene, partial sequence	Cryptosporidium parvum	383	383	100%	5e-102	100.00%	1250	KT151524.1
Cryptosporidium parvum isolate IQ.Cp-1 18S ribosomal RNA gene, partial sequence	Cryptosporidium parvum	383	383	100%	5e-102	100.00%	1352	KT151523.1
Cryptosporidium parvum Chr7_3pr-str genomic sequence	Cryptosporidium parvum	383	383	100%	5e-102	100.00%	52652	MZ892388.1
Cryptosporidium parvum Chr8_3pr-str genomic sequence	Cryptosporidium parvum	383	383	100%	5e-102	100.00%	60822	MZ892387.1
Cryptosporidium parvum Chr7_5pr-str genomic sequence	Cryptosporidium parvum	383	383	100%	5e-102	100.00%	59387	MZ892386.1
Cryptosporidium parvum isolate B12_34Da small subunit ribosomal RNA gene, partial sequence	Cryptosporidium parvum	383	383	100%	5e-102	100.00%	1208	OK310618.1
Cryptosporidium parvum chromosome 1	Cryptosporidium parvum	383	383	100%	5e-102	100.00%	894831	CP082119.1
Cryptosporidium parvum chromosome 2	Cryptosporidium parvum	383	383	100%	5e-102	100.00%	993129	CP082118.1

Description	Scientific Name	Max Score	Total Score	Query Cover	E value	Per. Ident	Acc. Len	Accession
Cryptosporidium parvum chromosome 7	Cryptosporidium parvum	383	766	100%	5e-102	100.00%	1346444	CP082113.1
Cryptosporidium parvum chromosome 8	Cryptosporidium parvum	383	383	100%	5e-102	100.00%	1355650	CP082112.1
Cryptosporidium parvum isolate IQN-No.2 small subunit ribosomal RNA gene, partial sequence	Cryptosporidium parvum	383	383	100%	5e-102	100.00%	724	MZ377025.1
Cryptosporidium parvum isolate IQN-No.1 small subunit ribosomal RNA gene, partial sequence	Cryptosporidium parvum	383	383	100%	5e-102	100.00%	724	MZ377024.1
Cryptosporidium parvum 18S ribosomal RNA gene, partial sequence	Cryptosporidium parvum	383	383	100%	5e-102	100.00%	1160	MT758442.1
Cryptosporidium parvum strain IOWA-ATCC chromosome 1	Cryptosporidium parvum	383	383	100%	5e-102	100.00%	920510	CP044422.1
Cryptosporidium parvum strain IOWA-ATCC chromosome 2	Cryptosporidium parvum	383	383	100%	5e-102	100.00%	992704	CP044421.1
Cryptosporidium parvum gene for 18S ribosomal RNA, partial sequence, isolate: Sakha212	Cryptosporidium parvum	383	383	100%	5e-102	100.00%	1728	AB513881.1
Cryptosporidium parvum gene for 18S ribosomal RNA, partial sequence, isolate: Sakha211	Cryptosporidium parvum	383	383	100%	5e-102	100.00%	1745	AB513880.1
Cryptosporidium parvum gene for 18S ribosomal RNA, partial sequence, isolate: Sakha210	Cryptosporidium parvum	383	383	100%	5e-102	100.00%	1656	AB513879.1
Cryptosporidium parvum gene for 18S ribosomal RNA, partial sequence, isolate: Sakha209	Cryptosporidium parvum	383	383	100%	5e-102	100.00%	1651	AB513878.1
Cryptosporidium parvum gene for 18S ribosomal RNA, partial sequence, isolate: Sakha208	Cryptosporidium parvum	383	383	100%	5e-102	100.00%	1725	AB513877.1
Cryptosporidium parvum gene for 18S ribosomal RNA, partial sequence, isolate: Sakha207	Cryptosporidium parvum	383	383	100%	5e-102	100.00%	1624	AB513876.1
Cryptosporidium parvum gene for 18S ribosomal RNA, partial sequence, isolate: Sakha206	Cryptosporidium parvum	383	383	100%	5e-102	100.00%	1620	AB513875.1
Cryptosporidium parvum gene for 18S ribosomal RNA, partial sequence, isolate: Sakha205	Cryptosporidium parvum	383	383	100%	5e-102	100.00%	1703	AB513874.1

Description	Scientific Name	Max Score	Total Score	Query Cover	E value	Per. Ident	Acc. Len	Accession
Cryptosporidium parvum gene for 18S ribosomal RNA, partial sequence, isolate: Sakha204	Cryptosporidium parvum	383	383	100%	5e-102	100.00%	1615	AB513873.1
Cryptosporidium parvum gene for 18S ribosomal RNA, partial sequence, isolate: Sakha203	Cryptosporidium parvum	383	383	100%	5e-102	100.00%	1602	AB513872.1
Cryptosporidium parvum gene for 18S ribosomal RNA, partial sequence, isolate: Sakha103	Cryptosporidium parvum	383	383	100%	5e-102	100.00%	1723	AB513858.1
Cryptosporidium parvum gene for 18S ribosomal RNA, partial sequence, isolate: Sakha201	Cryptosporidium parvum	383	383	100%	5e-102	100.00%	1706	AB513870.1
Cryptosporidium parvum gene for 18S ribosomal RNA, partial sequence, isolate: Sakha113	Cryptosporidium parvum	383	383	100%	5e-102	100.00%	1724	AB513868.1
Cryptosporidium parvum gene for 18S ribosomal RNA, partial sequence, isolate: Sakha112	Cryptosporidium parvum	383	383	100%	5e-102	100.00%	1628	AB513867.1
Cryptosporidium parvum gene for 18S ribosomal RNA, partial sequence, isolate: Sakha111	Cryptosporidium parvum	383	383	100%	5e-102	100.00%	1718	AB513866.1
Cryptosporidium parvum gene for 18S ribosomal RNA, partial sequence, isolate: Sakha110	Cryptosporidium parvum	383	383	100%	5e-102	100.00%	1689	AB513865.1
Cryptosporidium parvum gene for 18S ribosomal RNA, partial sequence, isolate: Sakha108	Cryptosporidium parvum	383	383	100%	5e-102	100.00%	1629	AB513863.1
Cryptosporidium parvum gene for 18S ribosomal RNA, partial sequence, isolate: Sakha107	Cryptosporidium parvum	383	383	100%	5e-102	100.00%	1742	AB513862.1
Cryptosporidium parvum gene for 18S ribosomal RNA, partial sequence, isolate: Sakha106	Cryptosporidium parvum	383	383	100%	5e-102	100.00%	1629	AB513861.1
Cryptosporidium parvum gene for 18S ribosomal RNA, partial sequence, isolate: Sakha105	Cryptosporidium parvum	383	383	100%	5e-102	100.00%	1726	AB513860.1
Cryptosporidium parvum gene for 18S ribosomal RNA, partial sequence, isolate: Sakha104	Cryptosporidium parvum	383	383	100%	5e-102	100.00%	1726	AB513859.1
Cryptosporidium parvum gene for 18S ribosomal RNA, partial sequence, isolate: Sakha102	Cryptosporidium parvum	383	383	100%	5e-102	100.00%	1625	AB513857.1
Cryptosporidium parvum isolate Izatnagar small subunit ribosomal RNA gene, complete sequence	Cryptosporidium parvum	383	383	100%	5e-102	100.00%	1744	EU660038.1

Description	Scientific Name	Max Score	Total Score	Query Cover	E value	Per. Ident	Acc. Len	Accession
Cryptosporidium parvum strain RPHN 18S ribosomal RNA gene, partial sequence	Cryptosporidium parvum	383	383	100%	5e-102	100.00%	1692	**DQ898158.1**
Cryptosporidium bovis isolate 94-1_2007 18S ribosomal RNA gene, partial sequence	Cryptosporidium bovis	383	383	100%	5e-102	100.00%	1732	**EF514234.1**
Cryptosporidium meleagridis strain AMHN 18S ribosomal RNA gene, partial sequence	Cryptosporidium meleagridis	383	383	100%	5e-102	100.00%	1610	**EF179381.1**
Cryptosporidium sp. azami 18S ribosomal RNA gene, partial sequence	Cryptosporidium sp. azami	383	383	100%	5e-102	100.00%	1749	**DQ520950.1**
Cryptosporidium sp. strain EGK 3 18S ribosomal RNA gene, partial sequence	Cryptosporidium sp.	383	383	100%	5e-102	100.00%	1803	**AF513227.2**
Cryptosporidium canis strain CPD1 18S ribosomal RNA gene, complete sequence	Cryptosporidium canis	383	383	100%	5e-102	100.00%	1741	**AF112576.1**
Cryptosporidium sp. CSP06 18S ribosomal RNA gene, complete sequence	Cryptosporidium sp. CSP06	383	383	100%	5e-102	100.00%	1743	**AF112573.1**
Cryptosporidium parvum strain K2 18S ribosomal RNA gene, complete sequence	Cryptosporidium parvum	383	383	100%	5e-102	100.00%	1750	**AF112570.1**
Cryptosporidium meleagridis strain CMEL 18S ribosomal RNA gene, complete sequence	Cryptosporidium meleagridis	383	383	100%	5e-102	100.00%	1744	**AF112574.1**
Cryptosporidium wrairi small subunit ribosomal RNA gene	Cryptosporidium wrairi	383	383	100%	5e-102	100.00%	1746	**U11440.1**
Cryptosporidium parvum strain CPF 18S ribosomal RNA gene, complete sequence	Cryptosporidium parvum	383	383	100%	5e-102	100.00%	1749	**AF112572.1**
Cryptosporidium parvum strain CPM1 18S ribosomal RNA gene, complete sequence	Cryptosporidium parvum	383	383	100%	5e-102	100.00%	1750	**AF112571.1**
Cryptosporidium parvum gene for 18S ribosomal RNA, partial sequence, isolate: Sakha109	Cryptosporidium parvum	383	383	100%	5e-102	100.00%	1746	**AB513864.1**
Cryptosporidium parvum strain Human C. parvum genotype (HFL5) small subunit ribosomal RNA gene, complete sequence	Cryptosporidium parvum	383	383	100%	5e-102	100.00%	1745	**AF093492.1**

Description	Scientific Name	Max Score	Total Score	Query Cover	E value	Per. Ident	Acc. Len	Accession
Cryptosporidium parvum strain CPRM1 18S ribosomal RNA gene, complete sequence	Cryptosporidium parvum	383	383	100%	5e-102	100.00%	1748	AF112569.1
Cryptosporidium parvum strain Human C. parvum genotype (HFL2) small subunit ribosomal RNA gene, complete sequence	Cryptosporidium parvum	383	383	100%	5e-102	100.00%	1750	AF093491.1
Cryptosporidium parvum 18S ribosomal RNA gene, complete	Cryptosporidium parvum	383	383	100%	5e-102	100.00%	1750	L16997.1
Cryptosporidium felis strain C8 18S ribosomal RNA gene, complete sequence	Cryptosporidium felis	383	383	100%	5e-102	100.00%	1781	AF112575.1
Cryptosporidium sp. EGK1 18S ribosomal RNA gene, partial sequence	Cryptosporidium sp. EGK1	383	383	100%	5e-102	100.00%	1748	AY237630.1
Cryptosporidium parvum clone OX1a 18S ribosomal RNA gene, partial sequence	Cryptosporidium parvum	383	383	100%	5e-102	100.00%	1698	AY204241.1
Cryptosporidium parvum clone B5b 18S ribosomal RNA gene, partial sequence	Cryptosporidium parvum	383	383	100%	5e-102	100.00%	1699	AY204240.1
Cryptosporidium parvum clone B2a 18S ribosomal RNA gene, partial sequence	Cryptosporidium parvum	383	383	100%	5e-102	100.00%	1699	AY204239.1
Cryptosporidium parvum clone HMa 18S ribosomal RNA gene, partial sequence	Cryptosporidium parvum	383	383	100%	5e-102	100.00%	1696	AY204238.1
Cryptosporidium parvum clone HMb 18S ribosomal RNA gene, partial sequence	Cryptosporidium parvum	383	383	100%	5e-102	100.00%	1696	AY204237.1
Cryptosporidium parvum clone OX2 18S ribosomal RNA gene, partial sequence	Cryptosporidium parvum	383	383	100%	5e-102	100.00%	1695	AY204236.1
Cryptosporidium parvum clone B5a 18S ribosomal RNA gene, partial sequence	Cryptosporidium parvum	383	383	100%	5e-102	100.00%	1698	AY204235.1
Cryptosporidium parvum clone B4a 18S ribosomal RNA gene, partial sequence	Cryptosporidium parvum	383	383	100%	5e-102	100.00%	1701	AY204234.1
Cryptosporidium parvum clone B1b 18S ribosomal RNA gene, partial sequence	Cryptosporidium parvum	383	383	100%	5e-102	100.00%	1701	AY204233.1

Description	Scientific Name	Max Score	Total Score	Query Cover	E value	Per. Ident	Acc. Len	Accession
Cryptosporidium parvum clone B2b 18S ribosomal RNA gene, partial sequence	Cryptosporidium parvum	383	383	100%	5e-102	100.00%	1698	AY204232.1
Cryptosporidium parvum clone B3b 18S ribosomal RNA gene, partial sequence	Cryptosporidium parvum	383	383	100%	5e-102	100.00%	1695	AY204231.1
Cryptosporidium parvum clone B3a 18S ribosomal RNA gene, partial sequence	Cryptosporidium parvum	383	383	100%	5e-102	100.00%	1701	AY204229.1
Cryptosporidium parvum clone B1a 18S ribosomal RNA gene, partial sequence	Cryptosporidium parvum	383	383	100%	5e-102	100.00%	1699	AY204227.1
Cryptosporidium canis 18S ribosomal RNA, complete sequence	Cryptosporidium canis	383	383	100%	5e-102	100.00%	1741	AB210854.1
Cryptosporidium parvum strain MT 18S ribosomal RNA gene, complete sequence	Cryptosporidium parvum	383	383	100%	5e-102	100.00%	1746	AF161856.1
Cryptosporidium parvum isolate KSU-1 small subunit ribosomal RNA gene, partial sequence	Cryptosporidium parvum	383	383	100%	5e-102	100.00%	1512	AF308600.1
Cryptosporidium sp. small subunit ribosomal RNA gene, partial sequence	Cryptosporidium sp.	383	383	100%	5e-102	100.00%	717	AF280053.1
Cryptosporidium parvum strain H7 18S ribosomal RNA gene, complete sequence	Cryptosporidium parvum	383	383	100%	5e-102	100.00%	1753	AF108865.1
Cryptosporidium parvum strain C1 18S ribosomal RNA gene, complete sequence	Cryptosporidium parvum	383	383	100%	5e-102	100.00%	1749	AF108864.1
Cryptosporidium M24 18S ribosomal RNA gene, complete sequence	Cryptosporidium sp. M24	383	383	100%	5e-102	100.00%	1753	AF108863.1
Cryptosporidium felis 18S ribosomal RNA gene, complete sequence	Cryptosporidium felis	383	383	100%	5e-102	100.00%	1784	AF108862.1
Cryptosporidium K1 18S ribosomal RNA gene, complete sequence	Cryptosporidium sp. K1	383	383	100%	5e-102	100.00%	1752	AF108860.1
Cryptosporidium parvum external transcribed spacer, partial sequence; 16S/18S ribosomal RNA gene, internal transcribed spacer 1, 5.8S ribosomal RNA gene and internal transcribed spacer 2, complete sequence; and 26S/28S subunit ribosomal RNA gene, partial sequence	Cryptosporidium parvum	383	383	100%	5e-102	100.00%	7820	AF040725.1

Description	Scientific Name	Max Score	Total Score	Query Cover	E value	Per. Ident	Acc. Len	Accession
Cryptosporidium sp. Sltl05c 18S ribosomal RNA gene, complete sequence	Cryptosporidium sp. Sltl05c	383	383	100%	5e-102	100.00%	1756	DQ295014.1
Cryptosporidium sp. Sbld05c 18S ribosomal RNA gene, complete sequence	Cryptosporidium sp. Sbld05c	383	383	100%	5e-102	100.00%	1755	DQ295013.1

Graphic Summary

Distribution of the top 101 Blast Hits on 100 subject sequences

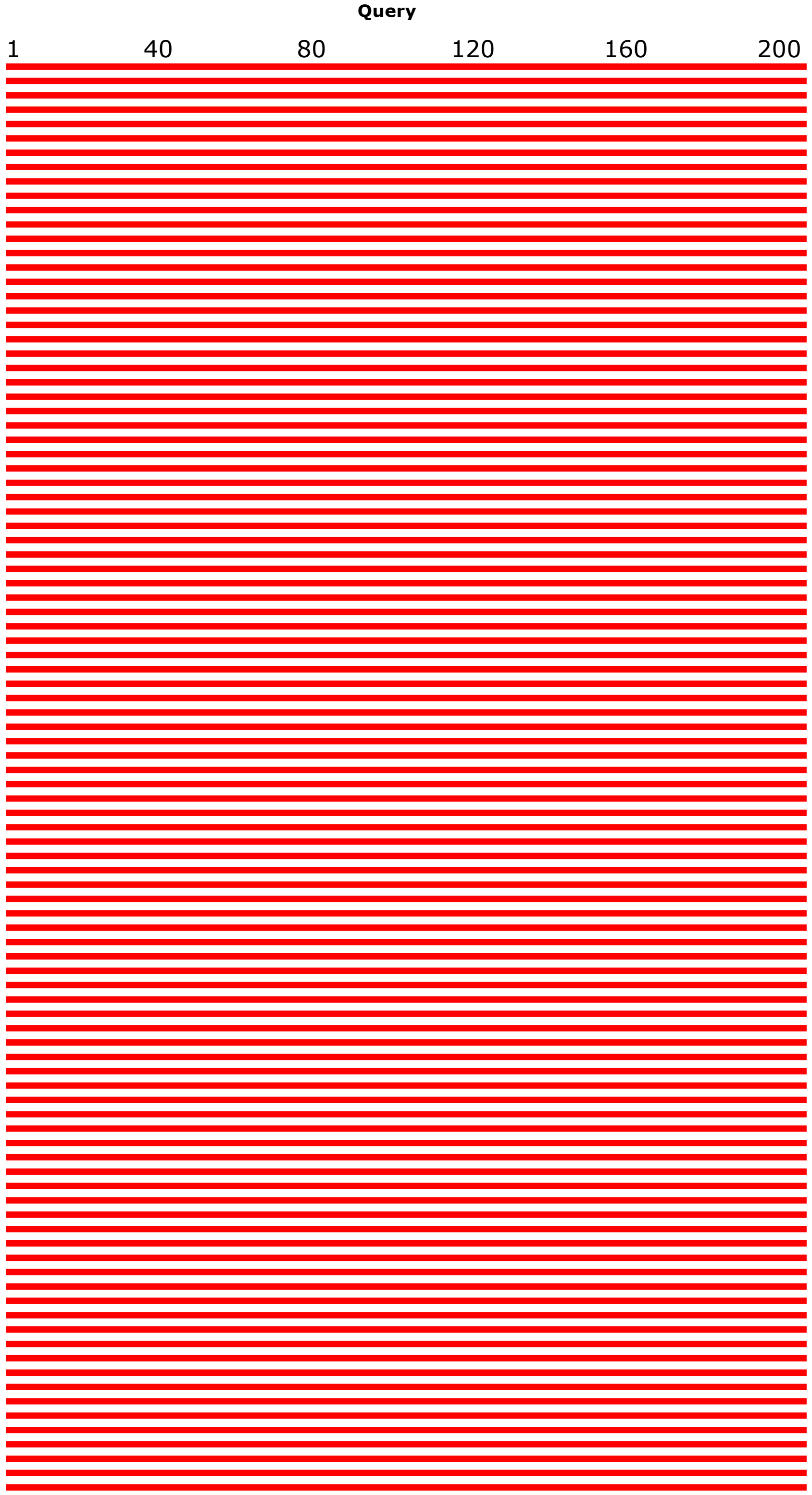

Alignments

Alignment view Pairwise CDS feature Restore defaults

Taxonomy

Reports

Top

F

Connect with NLM

National Library of Medicine
8600 Rockville Pike
Bethesda, MD 20894

Web Policies
FOIA
HHS Vulnerability Disclosure

Help
Accessibility
Careers

- NLM
- NIH
- HHS
- USA.gov
